# Hybrid Fixation for Syndesmotic Stabilisation: A Systematic Review of Clinical and Biomechanical Evidence

**DOI:** 10.3390/jcm15010107

**Published:** 2025-12-23

**Authors:** Jed Bailey, Richard Huynh, Konstantinos Tsikopoulos, Lyndon Mason, Vasileios Lampridis

**Affiliations:** 1 School of Medicine, University of Liverpool, Liverpool L69 7ZX, UK; hljbail3@liverpool.ac.uk (J.B.); vasileios.lampridis@liverpoolft.nhs.uk (V.L.); 2 Edinburgh Orthopaedics, Royal Infirmary of Edinburgh, Edinburgh EH16 4SA, UK; richard.huynh@nhs.scot; 3 Department of Trauma and Orthopaedics, North Bristol NHS Trust, Bristol BS10 5NB, UK; 4 Department of Trauma and Orthopaedics, Liverpool University Hospitals NHS Foundation Trust, Liverpool L7 8YE, UK

**Keywords:** syndesmotic injury, syndesmotic fixation, ankle fracture, hybrid fixation, screw fixation, dynamic fixation, clinical outcomes, biomechanical outcomes

## Abstract

**Background:** Syndesmotic injuries are a common type of ankle trauma, occurring in isolation or with fracture. Hybrid fixation (HF) combines screw and dynamic fixation, either as separate implants or within an integrated device, to stabilise such injuries. Despite clinical interest, no comprehensive evidence synthesis exists. This review evaluates clinical and biomechanical evidence on HF for syndesmotic stabilisation. **Methods**: EMBASE, Medline, the Cochrane Library, and PubMed databases were systematically searched until May 2025 to identify studies reporting HF in adults with syndesmotic injury. Clinical studies were appraised using the Methodological Index for Non-Randomised Studies (MINORS) and biomechanical studies using the Quality Appraisal for Cadaveric Studies (QUACS) tool. Given variation in HF configuration and outcome reporting, qualitative synthesis was performed in accordance with PRISMA 2020 guidelines. **Results**: Six studies were included: four clinical and two biomechanical. Across clinical studies, 93 patients received HF. Mean American Orthopaedic Foot and Ankle Society (AOFAS) scores, reported in two studies, were 93.3 at final follow-up. Radiographic outcomes indicated maintained syndesmotic reduction. Malreduction occurred in 3 patients (3.2%), unplanned implant removal in 3 patients (3.2%), and implant failure in 14 patients (15.1%). All implant failures were asymptomatic and confined to one study. Biomechanical studies demonstrated that HF restored native joint kinematics under simulated loading. **Conclusions**: Current evidence supports HF as an appropriate syndesmotic fixation strategy. However, methodological limitations of the available evidence, including observational design and variable follow-up durations, should be considered. Heterogeneity in construct design, inconsistent outcome reporting, and limited comparative research complicate interpretation. Future research should prioritise standardised outcome reporting and longer follow-up to thoroughly evaluate HF.

## 1. Introduction

Syndesmotic injuries are a common subset of ankle trauma [1,2,3]. Injury to the syndesmosis represents 1–11% of ankle injuries and occurs concomitantly in 10–23% of ankle fractures [4,5,6,7,8,9]. Inadequate diagnosis and treatment can result in chronic ankle instability, impaired mobility, and post-traumatic osteoarthritis [10,11]. Accurate reduction and appropriate stabilisation are critical to restore joint congruency and prevent long-term morbidity.

Screw fixation (SF) has been considered the standard stabilisation technique [12,13]. However, rigid fixation of the syndesmosis is not physiologic, may impair early weightbearing, and is associated with screw loosening or breakage [14,15,16]. Additionally, optimal screw configuration remains debated. Dynamic fixation (DF) using suture-button devices is a popular alternative. DF maintains reduction and permits micromotion, enabling earlier weightbearing and lower implant removal rates [17,18,19,20]. However, skin irritation, infection, and recurrent diastasis have been reported [21,22,23,24].

Comparative evidence of SF and DF is inconclusive. Several systematic reviews and meta-analyses favour DF for superior functional outcomes and fewer complications [25,26,27,28,29,30,31]. Others report comparable outcomes [24,32]. This uncertainty has prompted interest in hybrid fixation (HF) as an alternative. HF combines SF and DF, either as separate implants or within an integrated device. This dual modality approach offers stability and preservation of physiologic motion. Interest in HF is growing; an international survey reported HF was preferred by 18% of surgeons, following DF (47.1%) and SF (29.6%) [33].

HF is an emerging strategy with a limited but growing evidence base reflecting early clinical uptake [34,35,36,37,38,39]. Published studies demonstrate heterogeneity. Both integrated devices and separate implants have been evaluated, with differences in cortical engagement. Outcome reporting varies in the functional measures used and the timing of assessment. Postoperative protocols also differ with respect to weightbearing progression and implant retention or removal. Despite this variation, clinical data suggests maintained reduction and comparable functional outcomes to established techniques [34,35,36,37]. Further, biomechanical studies demonstrate restoration of native joint kinematics, supporting construct validity [38,39].

No comprehensive synthesis of evidence exists. This systematic review aims to evaluate current clinical and biomechanical evidence on HF for syndesmotic injury and defines priorities for future research.

## 2. Methods

This systematic review was conducted in accordance with the Preferred Reporting Items for Systematic Reviews and Meta-Analyses (PRISMA) 2020 statement [40]. The completed PRISMA 2020 checklist is provided in the Supplementary Materials. A protocol outlining objectives and eligibility criteria was developed and uploaded to the PROSPERO registry prior to study commencement (CRD420251109863).

### 2.1. Search Methodology

A systematic search was performed across “EMBASE”, “Medline”, “the Cochrane Library” and “PubMed” between 13 May 2025 and 27 May 2025 for completed published studies. Grey literature was not searched. Results were restricted to English-language publications, with no restrictions applied to publication date. Reference lists of included articles were manually screened to identify additional eligible studies. The search strategy combined keywords and Medical Subject Headings (MeSH) related to syndesmotic injury, hybrid fixation, and clinical or biomechanical outcomes. Boolean operators were used to link search terms. The complete search strategy for each database is detailed in Table 1.

### 2.2. Eligibility Criteria

Studies reporting HF for syndesmotic ankle injuries in adult populations were included. HF was defined as a fixation configuration combining a syndesmotic screw and dynamic fixation device, either as separate implants or integrated within a single system. Exclusion criteria were as follows: (1) case reports; (2) conference abstracts without full text; (3) technical notes or expert opinions; (4) articles reporting ankle syndesmosis fixation techniques other than hybrid fixation.

### 2.3. Screening

EndNote 21.4 (Clarivate Analytics) was used to manage reference [41]. Duplicates were identified using EndNote’s automated duplicate detection and removed manually following review of title, authors, year, journal and DOI/PMID fields where available. Two reviewers (J.B., R.H.) independently screened titles and abstracts. Consensus was sought prior to full-text review. Discrepancies were resolved by a third reviewer (V.L.). Final inclusion was determined following full text assessment of articles meeting eligibility criteria.

### 2.4. Data Extraction and Outcomes of Interest

Data were extracted independently by one reviewer (J.B.) using a standardised form developed in Microsoft Excel Version 16.102.2 (Microsoft Corporation, Redmond, WA, USA) [42]. Corresponding authors were not contacted for missing data.

For clinical studies, the following outcome data were extracted where available: main reported functional outcomes, radiographic parameters (MCS, TFO, TFCS), malreduction, unplanned implant removal, implant failure, complications (excluding implant failure and malreduction) and important findings. For biomechanical studies, data were extracted on testing materials, comparator interventions, protocol and variables if applicable.

### 2.5. Data Synthesis

Given the heterogeneity in HF construct configuration, study design, and variable outcome reporting, meta-analysis was not feasible. Therefore, a qualitative synthesis of findings was completed.

### 2.6. Quality Assessment

Clinical studies were appraised using the Methodological Index for Non-Randomised Studies (MINORS) [43]. This instrument validates the methodological quality across 12 items. Items are scored 0 if not reported; 1 when reported but inadequate; and 2 when reported and adequate. In comparative clinical studies, the maximum score is 24; for non-comparative studies, the maximum score is 16 [43]. For non-comparative studies, overall methodological quality was defined as good for scores of 15–16; moderate for scores of 9–14; and poor for scores < 8. For comparative studies, methodological quality was defined as good for scores of 23–24; moderate for scores of 15–22; and poor for scores < 14.

Cadaveric biomechanical studies were evaluated using the Quality Appraisal for Cadaveric Studies (QUACS) score [44]. This tool evaluates methodological quality of cadaveric studies across 13 items. Each item is assigned a score of 1, indicating the criterion is met, or 0 if the criterion is not met. Good methodological quality was defined as a score > 10.

No studies were excluded by quality assessment results. Instead, quality assessment informed interpretation of the evidence, with greater caution applied to findings with lower methodological scores.

## 3. Results

**Figure 1:**
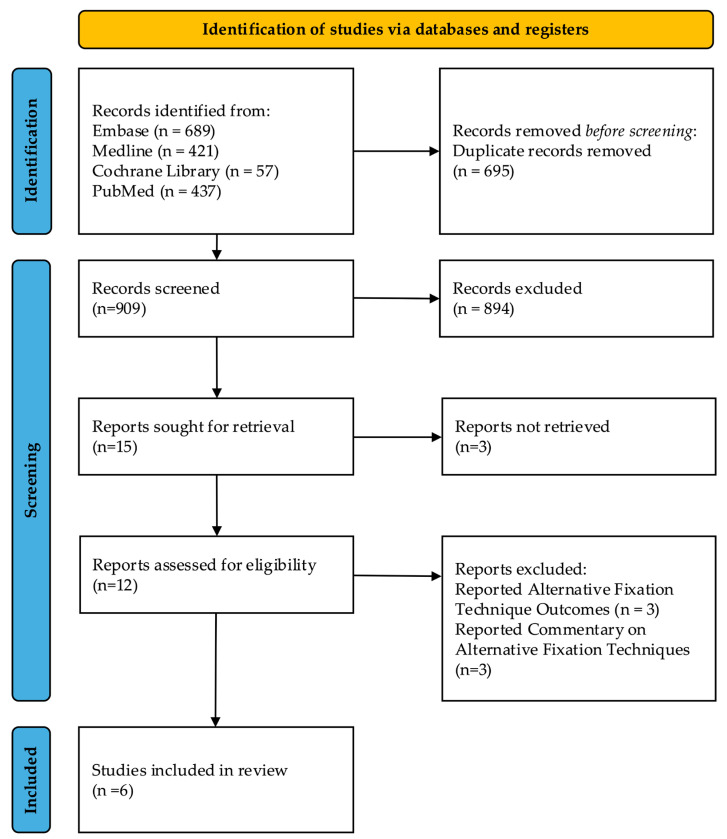
PRISMA flow diagram summarising study selection process. PRISMA, Preferred Reporting Items for Systematic Reviews and Meta-Analyses.

The preliminary search identified 1604 articles. After duplicate removal, 909 unique records underwent title and abstract screening. A total of 894 articles, which did not meet predefined inclusion criteria or were not relevant to the research topic, were excluded. Following the initial screening, 15 articles were selected for full-text review. Three articles were unavailable, as they were conference abstracts only. After thorough evaluation of the 12 remaining articles, 6 were excluded for reasons including reporting alternative fixation techniques to HF or not reporting extractable outcome data on HF. Ultimately, six articles published between 2013 and 2024 were included: four clinical studies and two biomechanical studies [34,35,36,37,38,39]. It should be noted that the limited number of included studies reflects the early clinical uptake of HF and prespecified eligibility criteria requiring explicit evaluation of hybrid constructs with extractable clinical or biomechanical outcome data. The article selection process is summarised in Figure 1.

### 3.1. Quality Assessment of Clinical Studies

**Figure 2:**
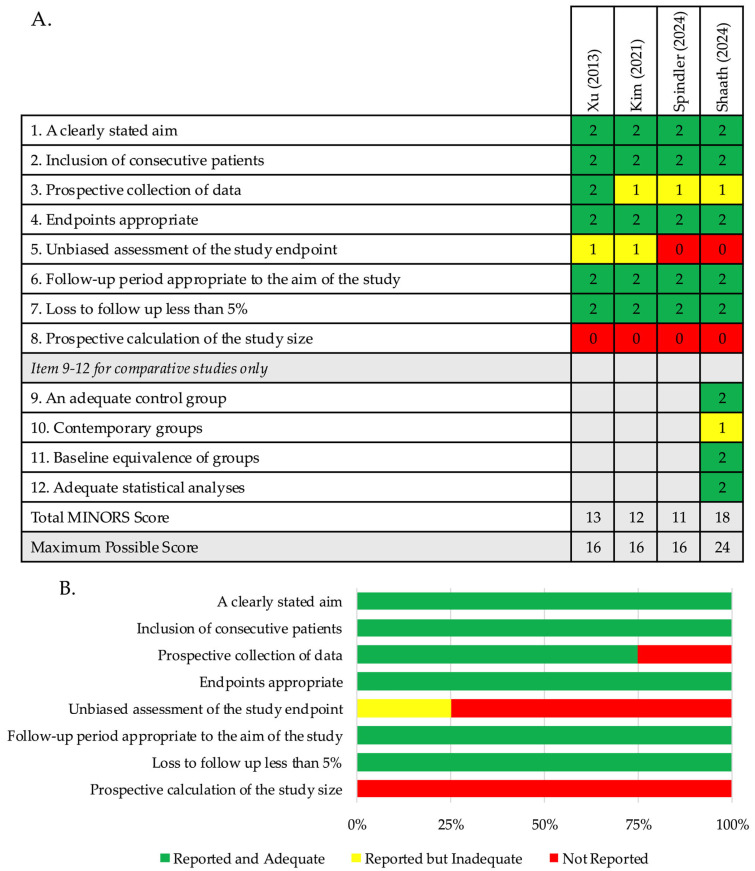
Methodological quality assessment of clinical studies: (**A**) The score for each MINORS domain of each included clinical study; (**B**) The weighted bar plot displays the distribution of scores across items 1–8 on the MINORS criteria [34,35,36,37].

Four clinical studies were included: three retrospective studies and one prospective study. One study was a comparative intervention study, with a MINORS score of 18 [34]. The remaining three studies were non-comparative, with an average MINORS score of 12 ± 0.1 [35,36,37]. Item-by-item scoring for each clinical study is detailed in Table 2. The overall results are summarised in Figure 2.

### 3.2. Clinical Results

Eight clinical studies underwent full-text review, and four were excluded. Lenz et al. did not report HF, instead reporting syndesmotic stabilisation with a single suture-button (TightRope; Arthrex Inc., Naples, FL, USA) and an Internal Brace with Fibretape (Arthrex Inc., Naples, FL, USA) over the anterior inferior tibiofibular ligament (AITFL) [45]. Three studies provided commentary on management techniques of syndesmotic injury without HF outcomes and were excluded [46,47,48].

Four studies (level of evidence III and IV) published between 2013 and 2024 were included. A total of 93 patients with syndesmotic injury were treated with HF. One study reported immediate postoperative complications only [37]. Across remaining studies, mean follow-up for patients receiving HF was 16.2 months (range 3 to 24 months) [34,35,36].

All patients were aged over 18 years and underwent HF for syndesmotic ankle injuries. Construct configurations varied. Xu et al. used the “Assembled Bolt-Tightrope System” (ABTS) (Naton Medical Group Ltd., Beijing, China) [35]. This comprised a pre-cut bolt, nut, oblong button, and a 2-0 FiberWire suture (Arthrex, Naples, FL, USA) [35]. The bolt component consisted of a 4.0 mm tip, a 3.0 mm smooth rod, and a 3.5 mm threaded end, which accommodated the suture-button [35]. Shaath et al. used the “Novel Syndesmotic Repair Implant” (NSRI) (DePuy Synthes, West Chester, PA, USA) [34]. This comprised a tibial screw connected to a fibular anchor via a suture bridge, placed in the same anatomical location as a suture-button device, without breaching the medial tibial cortex [34]. Kim et al. used separate implants: a suture-button (TightRope, Arthrex, USA) and a 3.5 mm cortical screw (Synthes, USA) [36]. Spindler et al. used a single suture-button device (TightRope; Arthrex, USA) and a syndesmotic screw [37]. Study characteristics are summarised in Table 3.

Surgical techniques were generally consistent, although procedure reporting varied. Three studies stated that operations were performed by senior orthopaedic surgeons [34,35,36]. Two studies specified that patients were positioned supine, and that a tourniquet control was utilised [35,36]. The type of anaesthesia—spinal anaesthesia—was reported in only one study [35]. Associated malleolar fractures were managed with open reduction and internal fixation (ORIF) before syndesmotic stabilisation in all studies [34,35,36,37]. Two studies assessed syndesmotic integrity intraoperatively, using external rotation and hook tests and external rotation and lateral stress tests, respectively [34,36]. Reduction clamps were used to achieve syndesmotic reduction, where diastasis persisted following fixation, in three studies [35,36,37].

Rehabilitation protocols varied, but followed progression from non-weightbearing to full weightbearing. Xu et al. reported immobilisation in a non-weightbearing below-knee cast for 2 weeks. Patients with stable fracture fixation and satisfactory wound healing progressed to partial weightbearing in a below-knee walking cast or walker boot 2 weeks postoperatively. If fixation was unstable, non-weightbearing continued 4 further weeks, with full weightbearing permitted 6 weeks postoperatively [35]. Kim et al. reported that patients with medial malleolar fractures were managed with removable short leg splint for 4 weeks. Patients with deltoid ligament injuries were immobilised in a short leg cast for the same duration. In all cases, partial weightbearing began at 4 weeks, with full weightbearing at 6 weeks [36]. Shaath et al. maintained non-weightbearing for 6 weeks following surgery. Patients with isolated ligamentous injuries were non-weightbearing for 3 months [34]. Spindler et al. reported incidence of malreduction immediately postoperatively, and did not report a rehabilitation protocol [37]. One study reported routine hardware removal at 12 months [35].

Two studies reported the American Orthopaedic Foot and Ankle Society (AOFAS) scores, with average score 93.3 at final follow-up (range 12–36 months) [35,36]. One study reported the Olerud–Molander Ankle Score (OMAS) and Visual Analogue Scale (VAS) at final follow-up, of 85 and 1.7, respectively [36]. Radiographic parameters were reported variably [34,35,36]. The primary outcomes in the included comparative study were radiographic outcomes. No significant differences were reported between the HF group and DF group [34]. Of 93 patients treated with HF, malreduction occurred in 3 cases, unplanned implant removal in 3 cases, implant failure in 14 cases, and other postoperative complications in 0 cases [34,35,36,37]. One study reported routine hardware removal, which was excluded from the implant removal summary [35]. All cases of implant failure were clinically asymptomatic [36]. A summary of reported outcome measures is shown in Table 4 and Table 5.

### 3.3. Quality Assessment of Biomechanical Studies

**Figure 3:**
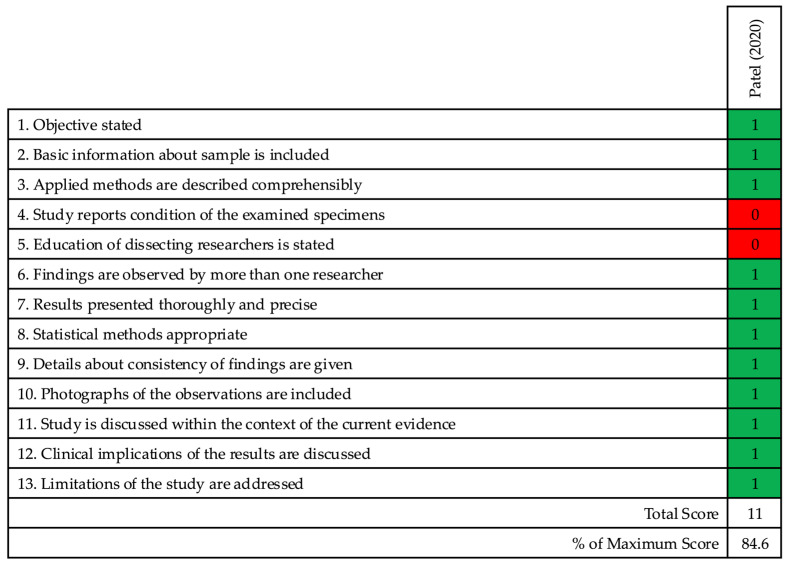
Methodological quality assessment of the included cadaveric biomechanical study. Results are presented using the Quality Appraisal for Cadaveric Studies (QUACS) tool [38].

The following two biomechanical studies were included: one cadaveric study [38] and one finite element analysis (FEA) study [39]. The cadaveric study was appraised using the QUACS tool, scoring 11 out of 13. Item-by-item scoring is shown in Table 6. The results are summarised in Figure 3.

### 3.4. Biomechanical Results

Four biomechanical studies were identified for full-text review, and two were excluded. Chuzhak et al. reported a “stable-elastic fixation” combining a fibula intramedullary nail with a suture-button device, rather than HF [49]. Goetz et al. reported a “prototype structurally augmented flexible trans-syndesmotic fixation device” combining a suture-button-like element and metal sleeve, rather than HF [50].

Two studies (level of evidence V) met inclusion criteria. One was a cadaveric study, using nine fresh-frozen lower leg specimens, published in 2020 [38]. The other was a finite element analysis published in 2023 [39]. A summary of construct configurations, testing protocols, and key findings is provided in Table 7.

Both studies evaluated the extent to which HF constructs restored native joint kinematics compared with the intact model [38,39]. Both utilised a 3.5 mm syndesmotic screw. Patel et al. used tricortical screw fixation with Invisiknot device (Smith & Nephew, Memphis, TN, USA) [38]. Mercan et al. modelled quadricortical screw fixation with No.5 FiberWire suture (Arthrex, USA) [39]. Mercan et al. evaluated two HF constructs with implants positioned 2 cm or 4 cm proximal to the tibial plafond: Hybrid-1 consisted of the screw at 4 cm and suture-button at 2 cm; Hybrid-2 consisted of the screw at 2 cm and suture-button at 4 cm [39].

Assessed parameters included lateral and posterior fibular translation relative to the tibia and external rotation of the fibula. Patel et al. reported in response to axial compression and inversion torque, HF reduced lateral fibular translation, and external fibular rotation at 15° plantar flexion (*p* < 0.05) [38]. However, overconstraint of motion during inversion was noted [38]. Mercan et al. reported the hybrid-1 construct and SF most similarly restored native joint kinematics in posterior translation and external rotation [39].

## 4. Discussion

Functional outcomes were inconsistently reported across included clinical studies. Two studies reported AOFAS score, with a mean score of 93.3 at final follow-up (range 12–36 months) [35,36]. One study additionally reported AOFAS scores at 12 months follow-up, with a mean of 95.4 [35]. Previous systematic reviews comparing SF and DF techniques have reported AOFAS scores at 12 months follow-up. Elabd et al., in their review of eight clinical studies, of which five were randomised controlled trials, reported average AOFAS scores of 92.4 and 86.6 for DF and SF, respectively [32]. Zhang et al. reported similar results, with an average of 91.1 for the DF group, and 87.8 for the SF group, in their review of nine studies, including three randomised controlled trials [30].

Available data on HF demonstrates comparable or superior functional outcomes. However, interpretation and comparison are limited by small sample sizes and variation in follow-up duration. Comparison with alternative fixation techniques is complicated by quality of evidence of included articles reporting AOFAS scores (level of evidence IV) compared to randomised controlled trials reporting SF and DF. It should also be acknowledged that there is criticism of the AOFAS score because of the ceiling effect and combination of patient-reported and clinician measured outcomes [51]. Despite limitations, functional outcomes support HF as an appropriate fixation strategy.

All clinical studies reported adverse events following HF. Malreduction occurred in 3.2%, unplanned implant removal in 3.2%, and implant failure in 15.1% of patients, with no other postoperative complications recorded. Previous systematic reviews comparing SF and DF reported malreduction rates of 5.9–12.6% and 1–2.5%; implant removal rates of 40.2–42.4% and 3.7%; implant failure rates of 26.8–30.9% and 0%; and complication rates of 12.3% and 7.6%, respectively [30,32]. This evidence suggests that HF constructs have a comparable complication profile. It should be noted all implant failures were reported by Kim et al., who used separate screw and suture-button components [36]. No cases of failure were reported with integrated devices. Kim et al. observed screw loosening in 78.6% of cases and screw breakage in 21.4%, identified radiographically at follow-up and without clinical symptoms [36]. This raises an important question regarding the clinical relevance of such events in a dual-component HF construct, given adequate functional outcomes and absence of late diastasis. The authors suggest that separate components may offer stability benefits [36]. These findings may indicate redundancy of the screw component once syndesmotic reduction has been achieved.

Unplanned implant removal was observed exclusively in the work by Shaath et al., although it was unclear whether the hybrid construct had failed [34]. It should be noted malreduction was only reported by Spindler et al. and Kim et al.; however, definitions differed [36,37]. Spindler et al. defined malreduction as measurements that differed between injured and uninjured sides beyond normal physiological limits [52,53]. Kim et al. defined syndesmotic malreduction based on the methods of Gardner et al. [54] and Naqvi et al. [55], which were a difference of 2.0 mm between the anterior and posterior tibiofibular distances and a difference greater than 2.0 mm between the posterior tibiofibular distances on each side, respectively.

Although the complication profile of HF demonstrates few clinically significant complications, comparison with SF and DF remains challenging. Heterogeneity in construct design and postoperative protocols, as well as lack of high-quality comparative evidence, limits conclusions regarding adverse events. Additionally, long-term complication risk remains uncertain, due to the short follow-up durations in available studies.

Radiographic measures were reported in three included studies [34,35,36]. Two studies reported MCS and TFO at final follow-up, with mean values of 3.45 mm and 8.15 mm, respectively [34,35]. Mean follow-up durations were 28 months [35] and 7 months, respectively [34]. TFCS was reported in two studies: one reported a postoperative value of 3.5 mm [36], and the other reported values of 4.1 mm at 12 month and final follow-up [35]. Syndesmotic diastasis has been defined in the literature as MCS > 4 mm, TFO < 6 mm, and TFCS >6 mm on anteroposterior radiograph [56,57,58]. All reported radiographic outcome values in included studies fall within these criteria, indicating that syndesmotic reduction was achieved. However, timing of radiographic assessment varied between the studies, limiting comparison of outcomes.

Postoperative protocols warrant discussion. Routine hardware removal remains debated in the literature. In SF specifically, routine removal has been associated with wound infection and recurrent diastasis [20,59,60]. In this review, only one study featured routine hardware removal, at 12 months [35]. However, the limited frequency of hardware removal in HF limits interpretation. Further evaluation of postoperative protocols is necessary, particularly regarding construct configuration, including whether removal is required in integrated systems or if selective removal is appropriate in constructs using separate screw and suture-button components.

Included biomechanical studies evaluated dual-component HF constructs, demonstrating such configurations appropriately restored native joint kinematics [38,39]. Mercan et al. noted that the screw provided resistance to rotational and posterior translation forces, with lateral fibular displacement managed by the suture-button. It has been reported in the literature that syndesmotic screws reduce coronal and sagittal translation [15,16]. The findings of Mercan et al. may suggest modification of the role of each component in an HF construct. Patel et al. demonstrated HF constructs closely restored native tibiofibular kinematics under varying loading conditions. These complementary results suggest that HF constructs may distribute physiological loads more effectively than isolated fixation approaches. However, Patel et al. noted overconstraint in inversion, which is of unknown clinical significance. Current biomechanical evidence supports the mechanical viability of HF, although conclusions are limited by the paucity of evidence.

Despite synthesising all available evidence, several limitations must be noted. Interpretation of findings should be considered in the context of study quality, as the evidence base consists predominantly moderate-quality studies. Further, the number of eligible studies was small; however, this reflects the current state of the HF literature, rather than limitations in the search or selection process. All clinical studies were observational, with small cohorts and variable follow-up durations, which limits generalisability. Eligibility criteria differed across studies, introducing further clinical heterogeneity. Construct configurations were inconsistent. Each included study evaluated HF with either integrated systems or separate devices. This complicates outcome comparison, and it remains unclear whether construct design influences outcomes. Postoperative weightbearing protocols varied in timeline, with immobilisation methods and criteria for progression reflecting uncertainty regarding optimal postoperative management. This variability limits comparison of functional and radiographic outcomes.

Considering conflicts of interest, one author in the Spindler et al. study reported consulting fees from Arthrex [37]. The study by Patel et al. was funded by the American Orthopaedic Foot and Ankle Society, with donated suture-button devices from Smith & Nephew [38]. Two authors reported receiving consulting fees, from Smith & Nephew and Zimmer Biomet, respectively [38].

Biomechanical studies demonstrated methodological constraints. The cadaveric study used older specimens, a static loading protocol, and non-randomised implant testing order [38]. The FEA model used by Mercan et al. was derived from a single subject, which may not reflect clinical variability [39]. These factors limit applicability to in vivo conditions.

## 5. Conclusions

HF represents a clinically relevant stabilisation strategy for syndesmotic injuries. Evidence supports maintenance of reduction and short- to mid-term functional outcomes comparable to established techniques, with a low rate of clinically significant complications. Biomechanical data support restoration of joint kinematics under simulated loading. However, the current literature remains insufficient to define optimal construct configuration or rehabilitation protocols. High-quality comparative studies directly comparing HF with SF or DF are needed, with standardised implant design, as well as surgical technique and postoperative protocols. Long-term follow-up is important to clarify construct durability, late complications, and specific indications. Future biomechanical research should use cyclic loading methodologies and varied injury patterns to strengthen translational relevance. Overall, HF can be considered a suitable alternative fixation strategy to SF and DF, in practice, until definitive evidence to inform best practice becomes available.

## Figures and Tables

**Table 1:** Database Search Strategies.

Database	Search Terms	Search Fields	Filters	Date of Search
EMBASE	(exp ankle injury/) OR (ankle* adj6 (injur* OR instabilit* OR laxity OR trauma* OR lesion* OR sprain* OR fracture* OR ruptur* OR broke* OR dislocat* OR syndesmo*)) OR “distal tibiofibular injur*” OR “distal tibiofibular joint*” OR “distal tibiofibular syndesmo*” OR “tibiofibular injur*” OR “tibiofibular syndesmo*” OR “high ankle sprain*” OR (syndesmo* adj3 (injur* OR ruptur* OR disrupt*)) OR “syndesmo*” OR (foot OR feet OR ankle*) OR exp foot/ OR exp ankle/ OR exp foot injury/ AND (“combined fixation” OR “dual fixation” OR “flexible fixation” OR “mixed fixation” OR “hybrid fixation” OR hybrid OR (screw* adj2 “suture button*”) OR (screw* adj2 tightrope*) OR (screw* adj2 “tight rope*”) OR “suture button construct*” OR “suturebutton construct*” OR “screw sutur*” OR “screwsutur*” OR “stable elastic” OR “elastic stable” OR (screw* adj2 endobutton*) OR (bolt* adj2 tightrope*) OR (bolt* adj2 “tight rope*”) OR “bolt-tightrope*” OR “tightrope-bolt*” OR “bolt* sutur*” OR “bolt-sutur*” OR “suture button-bolt*” OR (bolt* adj2 “suture button*”)) AND (“outcome*” OR “biomechanic* outcome*” OR exp treatment outcome/ OR (clinical adj2 outcome*) OR “clinical scor*” OR “functional scor*” OR “functional outcome*” OR “olerud-molander ankle scor*” OR “olerud-molander” OR exp complication/ OR “complication*” OR exp implant complication/ OR (implant* adj3 remov*) OR (implant* adj3 complication*) OR (implant* adj3 fail*) OR exp infection/ OR “malreduction” OR exp reoperation/ OR “reoperat*” OR “re-operat*” OR exp pain/ OR “pain*” OR (radiograph* adj3 outcome*) OR exp weight bearing/ OR “weight bear*” OR “weightbear*” OR exp joint dislocation/ OR “diastasis” OR (“American Orthopaedic Foot” adj2 “Ankle Society”))	Title, Abstract, Keywords	English Language	13 May 2025
Medline	exp Ankle Injuries/ OR (ankle* adj6 (injur* OR instabilit* OR laxity OR trauma* OR lesion* OR sprain* OR fracture* OR ruptur* OR broke* OR dislocatT OR syndesmoa)) OR “distal tibiofibular injurb” OR “distal tibiofibular joint*” OR “distal tibiofibular syndesmo*” OR “tibiofibular injurle” OR “tibiofibular syndesmo ” OR “high ankle sprain*” OR (syndesmo* adj3 (injur* OR ruptur* OR disrupt*)) OR “syndesmo*” OR (foot OR feet OR ankle*) OR exp Foot/ OR exp Ankle/ OR exp Foot Injuries/ AND (“combined fixation” OR “dual fixation” OR “flexible fixation” OR “mixed fixation” OR “hybrid fixation” OR hybrid OR (screw* adj2 “suture button*”) OR (screw* adj2 tightrope*) OR (screw* adj2 “tight rope*”) OR “suture button construct*” OR “suturebutton construct*” OR “screw sutur*” OR “screwsutur*” OR “stable elastic” OR “elastic stable” OR (screw* adj2 endobutton*) OR (bolt* adj2 tightrope*) OR (bolt* adj2 “tight rope*”) OR “bolt-tightrope*” OR “tightrope-bolt*” OR “bolt* sutur*” OR “bolt-sutur*” OR “suture button-bolt*” OR (bolt* adj2 “suture button*”)) AND (“outcome*” OR “biomechanic* outcome*” OR exp Treatment Outcome/ OR (clinical adj2 outcome*) OR “clinical scor*” OR “functional scor*” OR “functional outcome*” OR “olerud-molander ankle scor*” OR “olerud-molander” OR exp Postoperative Complications/ OR “complication*” OR (implant* adj3 remov*) OR (implant* adj3 complication*) OR (implant* adj3 fail*) OR exp Infections/ OR “malreduction” OR exp Reoperation/ OR “reoperat*” OR “re-operat*” OR exp Pain/ OR “pain*” OR (radiograph* adj3 outcome*) OR exp Weight-Bearing/ OR “weight bear*” OR “weightbear*” OR exp Joint Dislocations/ OR “diastasis” OR (“American Orthopaedic Foot” adj2 “Ankle Society”))	Title, Abstract, Keywords	English Language	15 May 2025
the Cochrane Library	MeSH descriptor: (Ankle Injuries) explode all trees OR ((injur* OR instabilit* OR laxity OR trauma* OR lesion* OR sprain* OR fracture* OR ruptur* OR broke* OR dislocat* OR syndesmo*) NEAR/6 ankle*) OR (“distal tibiofibular” NEXT injur*) OR (“distal tibiofibular” NEXT joint*) OR (“distal tibiofibular” NEXT syndesmo*) OR (tibiofibular NEXT injur*) OR (tibiofibular NEXT syndesmo*) OR (“high ankle” NEXT sprain*) OR ((injur* OR ruptur* OR disrupt*) NEAR/3 syndesmo*) OR (syndesmo*) OR (foot OR feet OR ankle OR ankles) OR MeSH descriptor: (Foot) explode all trees OR MeSH descriptor: (Ankle) explode all trees OR MeSH descriptor: (Foot Injuries) explode all trees AND (“combined fixation” OR “dual fixation” OR “flexible fixation” OR “mixed fixation” OR “hybrid fixation” OR “hybrid” OR (screw* NEAR/2 “suture button”) OR (screw* NEAR/2 tightrope*) OR (screw* NEAR/2 “tight rope”) OR (“suture button” NEXT construct*)) AND (“outcome*” OR “biomechanic* outcome*” OR MeSH descriptor: (Treatment Outcome) OR “clinical outcome*” OR “clinical scor*” OR “functional scor*” OR “functional outcome*” OR “olerud molander ankle scor*” OR “olerud-molander” OR MeSH descriptor: (Postoperative Complications) OR “complication*” OR “implant removal” OR “implant complications” OR “implant failure” OR MeSH descriptor: (Infections) OR “malreduction” OR MeSH descriptor: (Reoperation) OR “reoperat*” OR “pain*” OR “radiographical outcomes” OR MeSH descriptor: (Weight-Bearing) OR “weight bear*” OR “weightbear*” OR MeSH descriptor: (Joint Dislocations) OR “diastasis” OR “American Orthopaedic Foot Ankle Society”)	Title, Abstract, Keywords	English Language	21 May 2025
PubMed	(“Ankle Injuries”(MeSH) OR “ankle injury” OR “ankle instability” OR “ankle laxity” OR “ankle trauma” OR “ankle lesion” OR “ankle sprain” OR “ankle fracture” OR “ankle rupture” OR “broken ankle” OR “dislocated ankle” OR “ankle syndesmosis” OR “distal tibiofibular joint” OR “tibiofibular syndesmo*” OR “high ankle sprain*” OR “syndesmosis injury” OR “syndesmo*” OR “Foot” OR “feet” OR “ankle*”) AND (“combined fixation” OR “dual fixation” OR “flexible fixation” OR “mixed fixation” OR “hybrid fixation” OR “hybrid” OR “screw suture button” OR “screw tightrope” OR “screw tight rope” OR “suture button construct*” OR “screw sutur*” OR “screwsutur*” OR “stable elastic” OR “elastic stable” OR “screw endobutton” OR “bolt tightrope” OR “bolt tight rope” OR “bolt tightrope*” OR “bolt suture button”) AND (“outcome*” OR “biomechanic* outcome*” OR “Treatment Outcome”(MeSH) OR “clinical outcome” OR “clinical scor*” OR “functional scor*” OR “functional outcome*” OR “olerud molander ankle scor*” OR “olerud-molander” OR “Postoperative Complications”(MeSH) OR “complication*” OR “implant removal” OR “implant complications” OR “implant failure” OR “Infections”(MeSH) OR “malreduction” OR “Reoperation”(MeSH) OR “reoperat*” OR “pain*” OR “radiographical outcomes” OR “Weight-Bearing”(MeSH) OR “weight bear*” OR “weightbear*” OR “Joint Dislocations”(MeSH) OR “diastasis” OR “American Orthopaedic Foot Ankle Society”)	Title, Abstract, Keywords	English Language	27 May 2025

**Table 2:** Methodological quality assessment of clinical studies.

	Xu (2013) [35]	Kim (2021) [36]	Spindler (2024) [37]	Shaath (2024) [34]
1. A clearly stated aim	2	2	2	2
2. Inclusion of consecutive patients	2	2	2	2
3. Prospective collection of data	2	1	1	1
4. Endpoints appropriate	2	2	2	2
5. Unbiased assessment of the study endpoint	1	1	0	0
6. Follow-up period appropriate to the aim of the study	2	2	2	2
7. Loss to follow up less than 5%	2	2	2	2
8. Prospective calculation of the study size	0	0	0	0
9. An adequate control group	NA	NA	NA	2
10. Contemporary groups	NA	NA	NA	1
11. Baseline equivalence of groups	NA	NA	NA	2
12. Adequate statistical analyses	NA	NA	NA	2
Maximum possible score	16	16	16	24
Total score	13	12	11	18
Overall methodological quality	Moderate	Moderate	Moderate	Moderate

Abbreviations: NA = Not Applicable

**Table 3:** Characteristics of clinical studies.

Study	Year	Level of Evidence	Study Design	Intervention	Patients (*n*)	Mean Age (Years)	Fixation Construct	Follow-Up (Months), Mean (Range)	Outcome Measures	MINORS Score
Xu et al. [35]	2013	IV	P	HF	12	39.5	Assembled Bolt-Tightrope System (ABTS)	28 (25–33)	1, 2, 3, 4, 5, 6, 7, 8	13
Kim et al. [36]	2021	IV	R	HF	14	37.2	1 screw and 1 suture-button	19.7 (12–36)	1, 4, 5, 6, 7, 8, 9, 0	12
Spindler et al. [37]	2024	IV	R	HF	8	NR	1 screw and 1 suture-button	NR	5	11
Shaath et al. [34]	2024	III	R	HF	59	47	Novel Syndesmotic Repair Implant (NSRI)	7 (3–24)	2, 3, 5, 6, 7, 8	18
				DF	52	41	1 suture-button			

Abbreviations: HF = hybrid fixation; DF = dynamic fixation; R = retrospective data collection; P = prospective data collection series; NR = not reported. Outcome measure key: 1 = American Orthopaedic Foot and Ankle Society (AOFAS) score; 2 = medial clear space (MCS); 3 = tibiofibular overlap (TFO); 4 = tibiofibular clear space (TFCS); 5 = malreduction; 6 = unplanned implant removal; 7 = implant failure; 8 = complications; 9 = Olerud–Molander Ankle Score (OMAS); 0 = Visual Analogue Scale (VAS).

**Table 4:** Outcome measurement comparison of clinical studies.

Study	Intervention	AOFAS Score		MCS (mm)			TFO (mm)			TFCS (mm)			OMAS	VAS
		12 Months	Final Follow-Up	Post-Op	12 Months	Final Follow-Up	Post-Op	12 Months	Final Follow-Up	Post-Op	12 Months	Final Follow-Up		
Xu et al. [35]	HF	95.4	96.3	NR	3.1	3.2	NR	8.4	8.4	NR	4.1	4.1	NR	NR
Kim et al. [36]	HF	90.3	NR	NR	NR	NR	NR	NR	NR	3.5	NR	NR	85	1.7
Spindler et al. [37]	HF	NR	NR	NR	NR	NR	NR	NR	NR	NR	NR	NR	NR	NR
Shaath et al. [34]	HF	NR	NR	3.6	NR	3.7	7.9	NR	7.9	NR	NR	NR	NR	NR
	DF	NR	NR	3.1	NR	3.2	8.3	NR	8.3	NR	NR	NR	NR	NR

Abbreviations: HF = hybrid fixation; DF = dynamic fixation; NR = not reported. AOFAS = American Orthopaedic Foot and Ankle Society score; MCS = medial clear space; TFO = tibiofibular overlap; TFCS = tibiofibular clear space; OMAS = Olerud–Molander Ankle Score; VAS = Visual Analogue Scale.

**Table 5:** Outcome measurement comparison of clinical studies, cont.

Study	Intervention	Malreduction (n)	Unplanned Implant Removal (n)	Implant Failure (n)	Complications (n)	Weightbearing Protocol	Important Findings	Routine Implant Removal (Y/N)
Xu et al. [35]	HF	0	0	0	0	Partial weightbearing at 2 weeks Normal weightbearing at 6 weeks	ABTS showed strong short- to mid-term functional outcomes with no complications	Y
Kim et al. [36]	HF	1	0	14	0	Partial weightbearing at 4 weeks Normal weightbearing at 6 weeks	HF showed strong functional outcomes; implant failure, but not clinically significant	N
Spindler et al. [37]	HF	2	NR	NR	NR	NR	Malreduction was observed in 25% of patients who received HF	NR
Shaath et al. [34]	HF	0	3	0	0	Weightbearing at 6 weeks for fractures, 3 months for ligamentous injuries	NSRI showed comparable radiographic outcomes compared to suture-button fixation with low complication rates	N
	DF	0	2	0	2			

Abbreviations: HF = hybrid fixation; DF = dynamic fixation; NR = not reported; ABTS = Assembled Bolt-Tightrope System; NSRI = Novel Syndesmotic Repair Implant.

**Table 6:** Methodological quality assessment of the cadaveric study.

	Patel (2020) [38]
1. Objective stated	1
2. Basic information about sample is included	1
3. Applied methods are described comprehensibly	1
4. Study reports condition of the examined specimens	0
5. Education of dissecting researchers is stated	0
6. Findings are observed by more than one researcher	1
7. Results presented thoroughly and precisely	1
8. Statistical methods appropriate	1
9. Details about consistency of findings are given	1
10. Photographs of the observations are included	1
11. Study is discussed within the context of the current evidence	1
12. Clinical implications of the results are discussed	1
13. Limitations of the study are addressed	1
Maximum possible score	13
Total score	11
Overall methodological quality	Good

**Table 7:** Characteristics of biomechanical studies.

Study	Year	Level of Evidence	Study Design	Testing Material	Hybrid Fixation Constructs	Comparison Intervention	Testing Protocol	Testing Variables	Key Findings
Patel et al. [38]	2020	V	Cadaveric	9 fresh-frozen human cadaveric ankles	Proximal 3.5 mm screw and distal suture-button	Intact Injured SSF DSF SDF DDF (divergent)	200 N axial compression with 5 Nm external rotation or inversion torque Early weightbearing simulated at: 0°, 10° dorsiflexion, and 15° and 30° plantarflexion	Lateral fibula translation (mm) Posterior fibula translation (mm) External fibula rotation (°)	HF with a suture-button and tricortical screw most closely restored ankle joint kinematics for early weightbearing Overconstraint of motion in inversion observed
Mercan et al. [39]	2023	V	Finite Element Analysis	Finite element model from CT of one male subject	Hybrid-1: Proximal 3.5 mm screw and distal suture-button Hybrid-2: Distal 3.5 mm screw and proximal suture-button	Intact Injured SSF-2 cm * SSF-4 cm * DSF SDF-2 cm * SDF-4 cm * DDF (parallel)	2352 N axial compression and 235 N anterior tangential forces applied at tibial plateau Ankle tested in neutral position	Lateral translation (mm) Posterior translation (mm) External fibula rotation (°) Von Mises stress (MPa)	In Hybrid-1 model, the screw controlled posterior fibular translation and external rotation with the suture-button controlling lateral fibular translation Hybrid-1 construct is a suitable treatment alternative for syndesmotic injuries

Abbreviations: SSF = single screw fixation; DSF = double screw fixation; SDF = single dynamic fixation; DDF = double dynamic fixation. * Distance in cm proximal to the tibial plafond.

## Data Availability

No new data were created or analysed in this study.

## Supplementary Materials

The following supporting information can be downloaded at: https://www.mdpi.com/article/10.3390/jcm15010107/s1. PRISMA 2020 Checklist [40].
